# Degeneration of oil bodies by rough endoplasmic reticulum (rER)-associated protein during seed germination in *Cannabis sativa* L.

**DOI:** 10.1093/aobpla/plad082

**Published:** 2023-12-13

**Authors:** Eun-Soo Kim, Joon-Hee Han, Kenneth J Olejar, Sang-Hyuck Park

**Affiliations:** Institute of Cannabis Research, Colorado State University-Pueblo, 2200 Bonforte Blvd. Pueblo, CO 81001-4901, USA; Institute of Biological Resources, Chuncheon Bioindustry Foundation, 32, Soyanggang-ro, Chuncheon-si, Gangwon-do 24232, Republic of Korea; Department of Chemistry, Colorado State University-Pueblo, 2200 Bonforte Blvd. Pueblo, CO 81001-4901, USA; Institute of Cannabis Research, Colorado State University-Pueblo, 2200 Bonforte Blvd. Pueblo, CO 81001-4901, USA

**Keywords:** Cannabis, cotyledon, degeneration, embryo, endosperm, germination, oil body, protein body, rER-associated protein, seed

## Abstract

Abstract. Oil bodies serve as a vital energy source of embryos during germination and contribute to sustaining the initial growth of seedlings until photosynthesis initiation. Despite high stability in chemical properties, how oil bodies break down and go into the degradation process during germination is still unknown. This study provides a morphological understanding of the mobilization of stored compounds in the seed germination of *Cannabis*. The achenes of fibrous hemp cultivar (*Cannabis sativa cv.* ‘Chungsam’) were examined in this study using light microscopy, scanning electron microscopy and transmission electron microscopy. Oil bodies in *Cannabis* seeds appeared spherical and sporadically distributed in the cotyledonary cells. Protein bodies contained electron-dense globoid and heterogeneous protein matrices. During seed germination, rough endoplasmic reticulum (rER) and high electron-dense substances were present adjacent to the oil bodies. The border of the oil bodies became a dense cluster region and appeared as a sinuous outline. Later, irregular hyaline areas were distributed throughout oil bodies, showing the destabilized emulsification of oil bodies. Finally, the oil bodies lost their morphology and fused with each other. The storage proteins were concentrated in the centre of the protein body as a dense homogenous circular mass surrounded by a light heterogeneous area. Some storage proteins are considered emulsifying agents on the surface region of oil bodies, enabling them to remain stable and distinct within and outside cotyledon cells. At the early germination stage, rER appeared and dense substances aggregated adjacent to the oil bodies. Certain proteins were synthesized within the rER and then translocated into the oil bodies by crossing the half membrane of oil bodies. Our data suggest that rER-associated proteins function as enzymes to lyse the emulsifying proteins, thereby weakening the emulsifying agent on the surface of the oil bodies. This process plays a key role in the degeneration of oil bodies and induces coalescence during seed germination.

## Teaser

Our study examines the microstructural characteristics of Cannabis seeds and the structural changes in cell storage organelles during germination using light microscopy, scanning and transmission electron microscopy. We provide detailed observations and experimental evidence of the localization and function of storage proteins and their associated enzymes during seed germination. The study also offers insights into the mechanisms underlying the degeneration and coalescence of oil bodies, which are vital processes in seed germination.

## Introduction

Seed plants accumulate nutritional sources such as protein, lipids and carbohydrates in the endosperm or cotyledon for germination and post-germinative growth of the seedlings. Photosynthetic sugars are often polymerized into starch in amyloplasts or converted into lipids and stored as lipid droplets (LDs) in seeds. LDs are present in all plant cell types, ranging from a few LDs per cell in leaves to thousands of LDs per cell in seeds (Kang *et al.* 2022). LDs are often referred to in the literature by various terms, for example, lipid bodies, oil bodies, oleosomes or spherosomes depending on the characteristics of the species (Hsieh and Huang 2004; Purkrtova *et al.* 2008; Sánchez-Albarrán *et al.* 2019; Zienkiewicz and Zienkiewicz 2020; Nazari *et al.* 2022; Guzha *et al.* 2023). These organelles are surrounded by a half membrane and characteristically integrated with specific structural proteins (Tzen *et al.* 1993; Thiam *et al.* 2013; Huang 2018; Ischebeck *et al.* 2020).

LDs are not merely energy storage organelles, but also dynamic structures involved in diverse cellular metabolisms like membrane remodelling, regulation of energy homeostasis, stress responses and coordination between different organelles (Choi *et al.* 2022; Bouchnak *et al.* 2023). In addition, they play crucial roles at key sites in the freezing tolerance of seeds, engaging in direct interaction with glyoxysomes for seedling lipid degradation and producing antifungal compounds in leaves (Shimada *et al.* 2018; Olzmann and Carvalho 2019).

Lipids found in oilseeds are composed of a hydrophobic core filled with triacylglycerols (TAG), which are the most common storage lipids (Quettier and Eastmond 2009; Horn *et al.* 2013; Goold *et al.* 2015). The structural proteins of LDs commonly contain three membrane proteins known as oleosin, caleosin and steroleosin (Hsiao and Tzen 2011; Chapman *et al.* 2012; Huang 2018; Shimada *et al.* 2018). They are anchored in the phospholipid monolayer by a hydrophobic α-helical hairpin domain with a proline knot, and the C- and N-termini face of the cytosol (Alexander *et al.* 2002; Capuano *et al.* 2007; Purkrtova *et al.* 2008).

Oleosins are the most abundant integral membrane proteins of LDs in oilseeds. Particularly, the lipid droplet-associated proteins stabilize the LDs and prevent the coalescence or aggregation of this organelle in mature seeds (Huang 1992, 1994; Miquel *et al.* 2014). Hence, they are important regulators of LD dynamics; their ubiquitination, extraction and proteasomal degradation precede LD breakdown (Deruyffelaere *et al.* 2015; D’Andrea 2016).

Storage oil mobilization usually begins with seed germination. As a carbon or energy source in the germinating seeds, storage oil contributes to providing free-fatty-acids released from TAG by lipase or sugars through free-fatty-acid degradation by β-oxidation with subsequent gluconeogenesis (Graham 2008; Hielscher *et al.* 2017). The pathway of storage lipid conversion to sugars was examined in germinating lupin seeds (Borek and Ratajczak 2010; Borek *et al.* 2017). Subsequently, all the storage compounds are remobilized during post-germinative growth (Babazadeh *et al.* 2012; Miray *et al.* 2021).

Recently, some researchers reported on the LD degradation system in plants (Farquharson 2018; Kretzschmar *et al.* 2018; Traver and Bartel 2023). Since LDs are strongly associated with the endoplasmic reticulum (ER) (Suzuki 2017), this association has been observed at the electron microscopy level in many organisms (Fujimoto *et al.* 2013; Ohsaki *et al.* 2017; Sui *et al.* 2018; Renne *et al.* 2020; Kang *et al.* 2022; Zhang *et al.* 2022). During germination and seedling establishment, glyoxysomal enzymes degrade oil bodies to release storage lipids in seeds (Graham 2008; Shimada *et al.* 2018). Peroxisome contains the triacylglycerol lipase SUGAR-DEPENDENT1. This lipase is associated with the surface of the peroxisomes, and it is translocated to the oil body surface during seedling establishment (Thazar-Poulot *et al.* 2015).

*Cannabis* seeds contain approximately 18–30 % protein, 30–40 % oil and 25–34 % carbohydrate (Leonard *et al.* 2020; Vasantha Rupasinghe *et al.* 2020). Much of the knowledge of LD function in plants comes from studies of oilseeds (Laibach *et al.* 2015; Pyc *et al.* 2017b; Chen *et al.* 2023). Despite the importance of storing fats, oils and wax in seeds, our knowledge of the specificities of lipid metabolism remains uncertain.

This study represents a fundamental step towards the morphological elucidation of the mobilization mechanism of storage compounds in seeds. This research aimed to determine (i) the structural characteristics of storage compounds in the cotyledons of *Cannabis*, (ii) the degradation pathway leading to the β-oxidation of storage oil during seed germination, and (iii) the relationship between storage organelles such as oil bodies and protein bodies in oilseeds.

## Materials and Methods

The achenes of fibre hemp cultivar (*Cannabis sativa cv.* ‘Chungsam’) obtained from Dangjin Agricultural Technology Centre (DATC), South Korea were used in this study. Dangjin area located in Chungcheong Province in South Korea (37°03ʹN, 126°51ʹE) provides favourable environmental conditions for high-quality hemp seeds. These achenes were collected from an approved farm by DATC 8 months prior. They were stored in a seed storage chamber of DATC at 4 °C. Twenty achenes were germinated for two days on sheets of filter paper moistened with sterile water in glass Petri dishes (150 mm × 20 mm) in an incubator with 65 % relative humidity and 20 °C under darkness (Blandinières *et al.* 2021). The embryo samples were obtained from the germinating seeds at various times in the growth phase; early (12h), middle (18 h) and late stage (24 h) after germination.

For light microscopy, the seeds were dissected with a razor under the stereoscopic microscope and fixed for 2 h in 2 % glutaraldehyde in 25 mM phosphate buffer, pH 7.2. After being rinsed with deionized water, they were post-fixed for 1 h in 2 % osmic acid and dehydrated with a graded ethanol series (50, 70, 80, 90, 95, 100 % ethanol). Then the samples were embedded in Spurr’s resin for 14 h and polymerized for 48 h at 60 °C. Semithin sections of 0.4 μm in thickness were cut on an ultramicrotome (Reichert Ultracut S, Leica, Germany) with glass knives and stained with toluidine blue-basic fuchsin. For the histochemical study, the fresh sections of the embryo tissue were touched on the slide glasses and stained with Sudan III, Alcian blue and Astra Blue. All the samples were observed and photographed using a light microscope (Axiophot II, Zeiss, Germany).

For scanning electron microscopy (SEM), the achenes were fixed in the same protocol described in the light microscopy sample preparation. Then the samples were transferred into isoamyl acetate. The samples were subjected to critical point drying with pressurized liquid carbon dioxide (Bioradical E3000, Bio-Rad, USA). The dried specimens were mounted on aluminium stubs, coated with gold-palladium in a sputter coater (JFC-1110E, JEOL, Japan), and photographed in a FE-SEM (JSM-6700F, JEOL, Japan) at 15 kV.

For TEM, the seeds were treated with the SEM fixation method described above. The materials were dehydrated with a graded ethanol series and replaced with propylene oxide. Subsequently, ultra-thin sections of 70 nm thickness were cut with a diamond knife (Micro Star SU-30, Ted Pella, USA) using an ultramicrotome (Reichert Ultracut S, Leica, Germany) and sections were collected on 300 mesh copper grids. The sections were stained for 20 min with 1 % uranyl acetate and for 10 min with 1 % lead citrate. Image acquisition was performed with a transmission electron microscope (JEM-2000 EX II, JOEL, Japan) at 80 kV.

## Results

**Figure 1.**
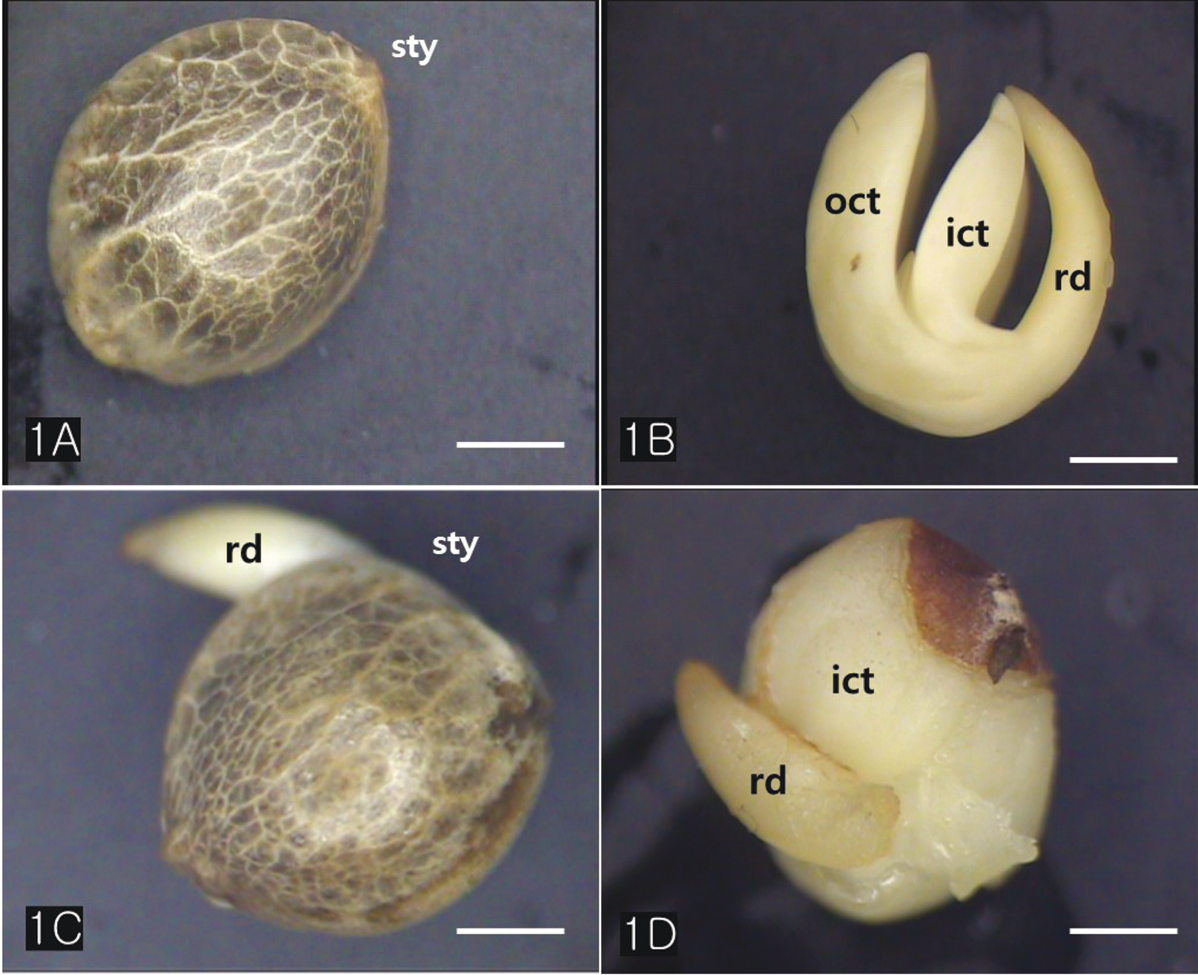
Light microscopy (LM) images of an entire achene and an extracted embryo. (A) A *Cannabis* achene. (B) The mature embryo is composed of two cotyledons and a radicle. The embryo is curved so that its axis is originally u-shaped, and the radicle end is adjacent to the distal pericarp. (C) The germinating seed showing emergence of a primary root. (D) The germinating *Cannabis* embryos. Abbreviations: ict; inner cotyledon, oct; outer cotyledon; rd; radicle, sty; style. Scale bars (A–F) = 2 mm.

**Figure 2.**
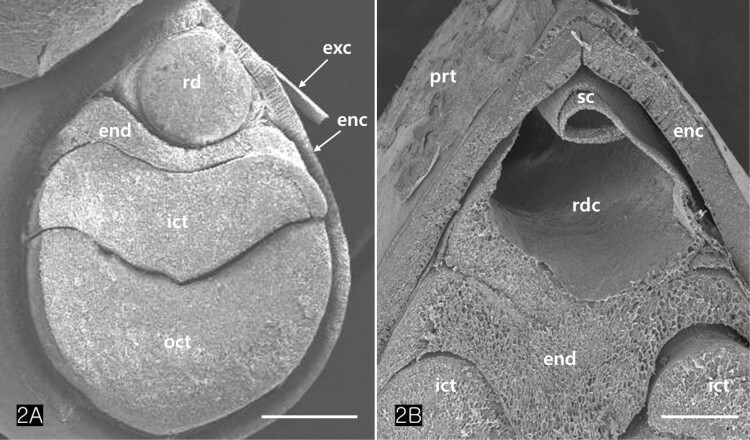
Scanning electron microscopy (SEM) images of an achene. (A) The seed contains two cotyledons, a radicle, and an endosperm between the internal cotyledon and the radicle. (B) Detail of a distal region of an achene. Note a radicle cavity and an endosperm showing tetrapodic appearance. Abbreviations: enc; endocarp, end; endosperm, exc; exocarp, ict; inner cotyledon, oct; outer cotyledon, prt; perianth, rd; radicle; rdc; radicle cavity, sc; seed coat. Scale bars (A) = 1 mm; (B) = 500 µm.

**Figure 3.**
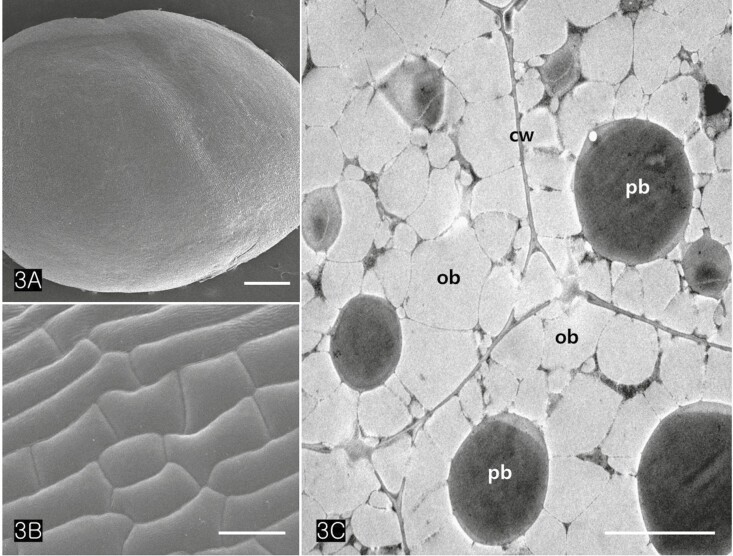
SEM and transmission electron microscopy (TEM) images of an embryo. (A) A mature *Cannabis* embryo (B) Highly magnified epidermal cells of the seed which consisted of rectangular cells with long and short lengths. (C) TEM image of cotyledonary cells bounded by unthickened primary walls illustrate storing numerous oil bodies and protein bodies in their cytoplasm. Abbreviations: cw; cell wall, ob; oil body, pb; protein body. Scale bars (A, C) = 500 µm; (B) = 25 µm.

*Cannabis* achenes have a hard pericarp encasing a single seed. In this study, the achenes varied in length from 4 to 5 mm, and in diameter from 3 to 4 mm (Fig. 1A and C). The seed consisted of an endosperm and an embryo with two cotyledons and a radicle (Fig. 1B and D). The axis of the *Cannabis* embryo was curved and contained a U-shaped feature (Fig. 1B). The tip of the radicle and cotyledons were oriented toward the stylar end of the achene (Fig. 1B and D). When the germination began, the radicle emerged from the pericarp at the stylar end and split the seed coats into halves that were attached at the base (Fig. 1C). The scanning electron micrographs of the *Cannabis* seed showed that it consisted of endosperm, two distinctive cotyledons (outer, and inner cotyledon) and a radicle in a piece of the embryo (Fig. 2A). Specifically, the endosperm was confined to a peripheral region between the inner cotyledon and radicle in the mature seed (Fig. 2B). The deshelled seed was smooth and oval or orbicular in form as well as the enclosed seed (Fig. 3A). The epidermal cells of the embryo were rectangular in shape and arranged end to end in rows. They were equal approximately 15 µm in width but differed in length, the longer one being 60 µm and the shorter 14 µm. (Fig. 3B). The cotyledonary cells, functioning as storage, contained numerous oil bodies and protein bodies (Fig. 3C).

**Figure 4.**
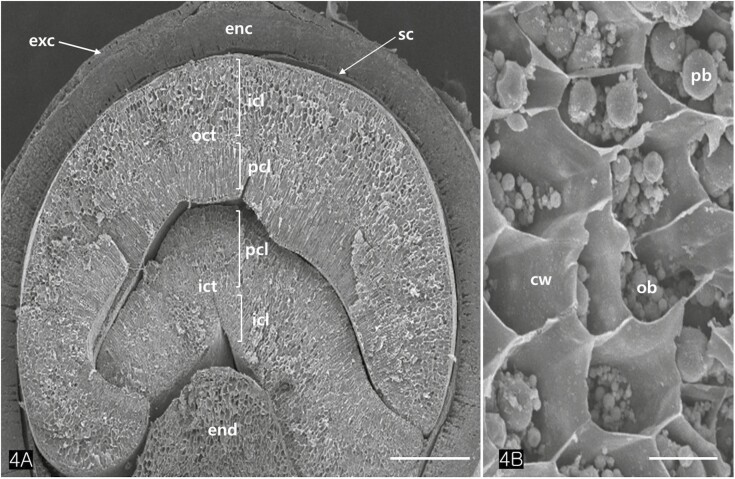
Transverse sectioned SEM images of an achene. (A) Two cotyledons consist of two distinct layers each, *i.e.,* the isodiametric cell layers (icl) and the palisade cell layers (pcl). (B) Close-up of the isodiametric cells. Abbreviations: cw; cell wall, enc; endocarp, end; endosperm, exc; exocarp, ict; inner cotyledon, ob; oil body, oct; outer cotyledon, pb; protein body, sc; seed coat. Scale bars (A) = 500 µm; (B) = 50 µm.

**Figure 5.**
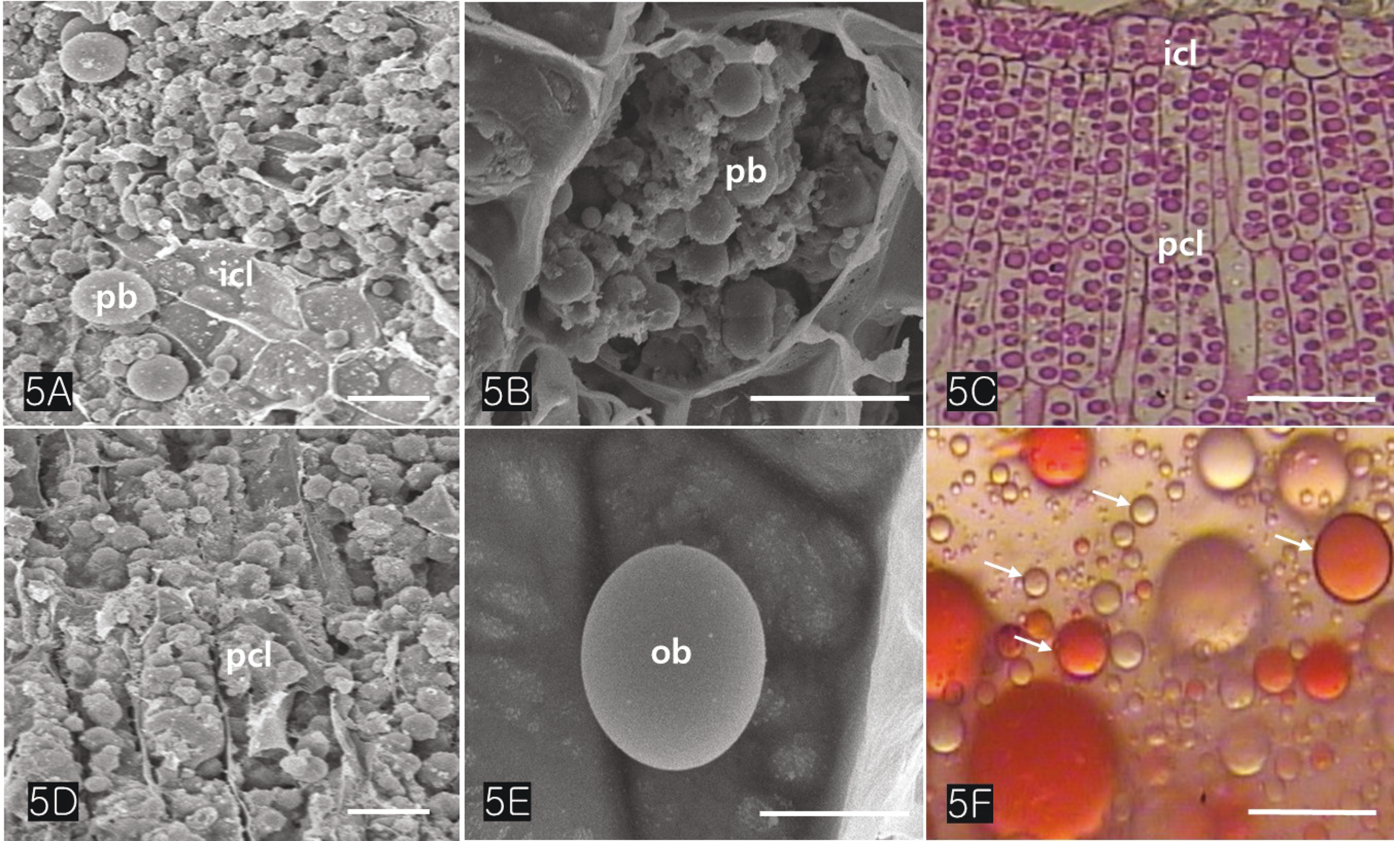
SEM and LM images of cotyledonary cells. (A) The large protein bodies and the small oil bodies can be distinguished. (B) An isodiametric cell of cotyledon illustrates a package of oil bodies and protein bodies. (C) LM image of cotyledon storage cells stained with toluidine bluebasic fuchsin. Each cotyledon consists of an isodiametric cell layer and a palisade cell layer. (D) The palisade cells containing both protein bodies and oil bodies are visible. (E) Close-up of an oil body in the cell. (F) The isolated oil bodies (arrows) stained with Sudan III are variable. Abbreviations: icl; isodiametric cell layer, ob; oil body, pcl; palisade cell layer, pb; protein body, sc; seed coat. Scale bars (A) = 50 µm; (B) = 10 µm; (C) = 50 µm; (D) = 10 µm; (E) = 5 µm; (F) = 2.5 µm.

The cotyledons comprised several layers of parenchymatous cells (isodiametric cells) and two or more layers of palisade cells (Fig. 4A). TEM images revealed that the protein bodies of cotyledon cells measured between 2.5 and 3.5 µm in diameter (Figs. 4C, 5A–C). Large protein bodies are surrounded by many oil bodies ranging from 0.7 to 1.8 µm in diameter. However, the size of the extracted oil bodies varied from 0.1 to 2 µm (Fig. 5F). Oil bodies in *Cannabis* seed appeared spherical and were sporadically distributed in the cells (Fig. 5D and E). Isolated oil bodies were obtained by smearing small pieces of cotyledon onto a microscope slide and staining them with Sudan III (Fig. 5F).

**Figure 6.**
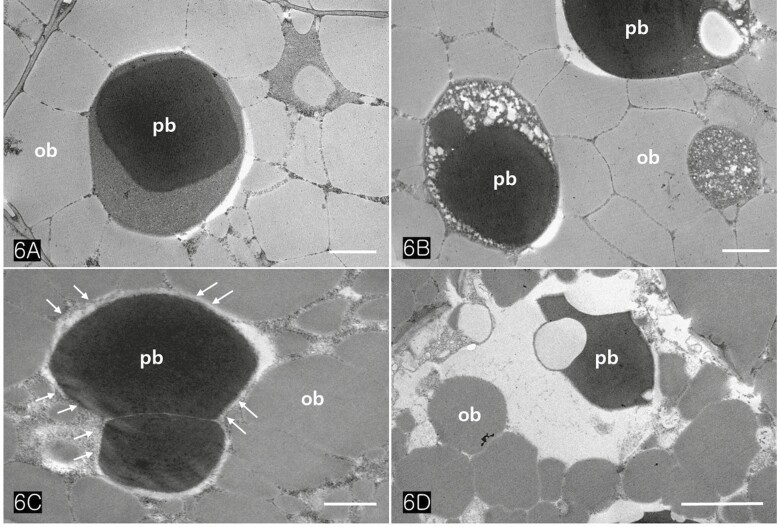
TEM images of cotyledonary cells showing degeneration of protein bodies during germination. (A) A protein body containing high electron-dense globoid and heterogeneous matrix and numerous oil bodies are packed in a storage cell. (B) Heterogeneous matrix of protein bodies is digested, forming a prominent enclave. (C) At the early stage of germination, oil bodies surrounding a protein body contribute to the degeneration of protein bodies (arrows). (D) Following the germination of the seed, protein bodies and lipid bodies are losing their shapes and electron densities in the cells. Note a large vacuole that develops after hydrolysis of the protein body. Abbreviations: ob; oil body, pb; protein body. Scale bars (A, B, and C) = 1 µm; (D) = 2 µm.

**Figure 7.**
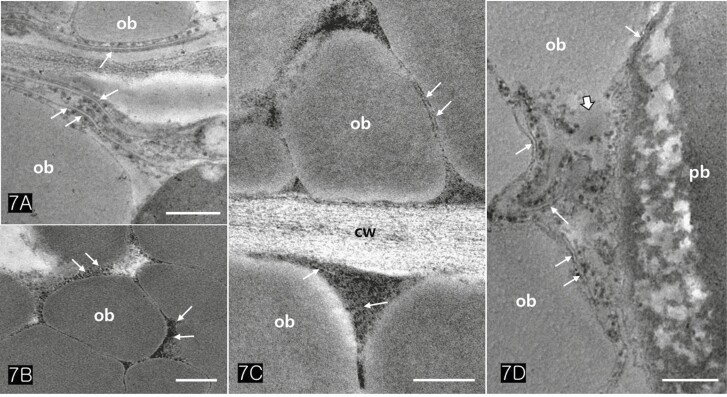
TEM images of cotyledonary cells illustrate the activity of rough ER. (A) Rough ER (arrows) was closely associated with oil bodies, reflecting the intimate functional relationship between the two organelles. (B, C) High electron-dense protein products (arrows) were aggregated outside of oil bodies. (D) Rough ER (arrows) directly acted to degenerate the membrane of oil bodies (wide arrow). Abbreviations: cw; cell wall, ob; oil body, pb; protein body. Scale bars (A, C) = 250 nm; (B) = 0.5 µm; (D) = 500 µm.

**Figure 8.**
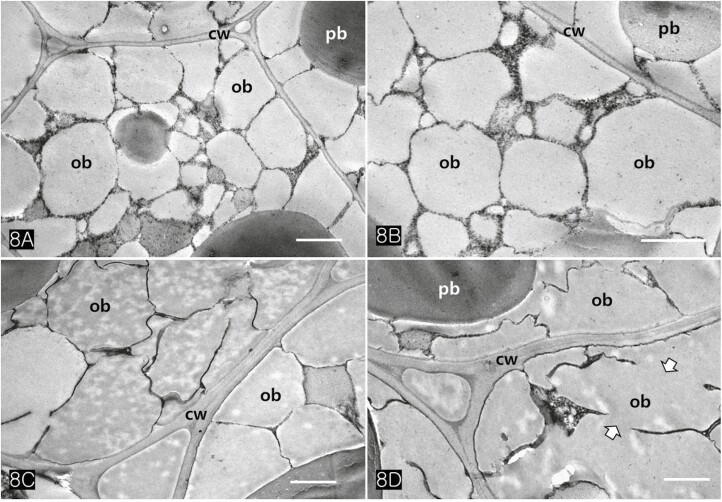
TEM images of cotyledonary cells showing degeneration of oil bodies during germination. (A, B) At the early stage of germination, dense materials aggregated adjacent to the oil bodies and associated with them. (C) At the middle stage, irregular hyaline areas were distributed throughout oil bodies, showing the destabilised emulsification of oil bodies. Note the border of oil bodies became a dense and sinuous outline. (D) The oil bodies lost their morphology and fused with one another at the late stage of germination (wide arrows). Abbreviations: cw; cell wall, ob; oil body, pb; protein body. Scale bars (A–D) =1 µm.

Protein bodies in *Cannabis* seeds contained electron-dense globoids with a heterogenous protein matrix. The storage proteins were concentrated in the centre of the protein body as a dense homogenous circular mass surrounded by a light heterogeneous area (Fig. 6A and B). As the major seed storage organelles in *Cannabis*, protein bodies and oil bodies within the cotyledon cells underwent unique morphological changes throughout germination (Fig. 6B–D). The protein bodies and oil bodies gradually degenerated in the cells and were used as a primary energy source during germination. During germination, the rER was frequently present in all cotyledon cells (Fig. 7A and D). As ribosomes and rER began to increase, dense substances were also concentrated in the outer region of oil bodies (Fig. 7B and C). At the early stage of germination, dense substances aggregated adjacent to the oil bodies and associated with them (Fig. 8A and B). Later, the border of the oil bodies became a dense cluster and appeared as a sinuous outline. In addition, irregular hyaline areas were distributed throughout the oil bodies, reflecting the destabilized emulsification of oil bodies (Fig. 8C). Finally, the oil bodies fused with one another and had an irregularly contoured surface (Fig. 8D).

## Discussion

Our fundamental data on seed germination provides insight into the understanding of the degradation mechanisms controlling the metabolism of storage proteins in the cotyledon of oilseeds. As small subcellular storage organelles, the protein bodies and oil bodies in the cotyledon cells of *Cannabis* seeds are gradually degenerated and used as a primary energy source during germination. Particularly, the biological function of storage proteins correlated with oil bodies stored in the cotyledon cells appears to be more diverse than simply constituting a source of carbon made available for the germinating seedling.

### Morphology of achenes in Cannabis

Even though some researchers have reported, there is still a lack of comprehensive studies on the structure of *Cannabis* fruits and seeds as they relate to hempseed-based food products (Oseyko *et al.* 2019; Farinon *et al.* 2020). Small (1974) described that domesticated *Cannabis* plants have large achenes longer than 3.7 mm and lack an adhering of the perianth. The fruits of uncultivated plants are small and possess an adhering perianth. These wild types of morphological characteristics such as smaller fruits, adhering perianth and an elongated base are more adaptive in a wild environment.

*Cannabis* achene varies in size and shape depending on the varieties and cultivars, the average length of fruit is reported from 2 to 6 mm, with diameters from 2 to 4 mm depending on diverse varieties and cultivars (Clarke 1981). In dry seeds, the outer cotyledon is remote from the radicle, whereas the inner cotyledon is adjacent to the radicle. The former is about 50 % heavier than the latter in *Cannabis* (Small and Antle 2008). Our result showed that the fibre type of achene was large ranging mostly 5 mm in length and 4 mm in diameter, and the perianth partially remained at the base.

Indehiscent dry fruit contains a single seed encased in a pericarp or fruit husk. Observation of the longitudinal and transverse sections of *Cannabis* achene revealed that the embryo was encased by a multi-layered pericarp and seed coat casing as shown in Fig. 2. The U-shaped embryo was distributed unevenly in the seed, with higher concentrations in the dorsoventral regions and lower concentrations in the two lateral sides, the radicle and the chalaza region. Both embryo and endosperm are derived from individual fertilization processes and develop while embedded in maternal tissues that form the seed coats, an outer protective layer (Walker *et al.* 2011).

In some species of Brassicaceae and Solanaceae, the endosperm is confined to a peripheral aleurone-like cell layer in the mature seed (Pabon-Mora and Litt 2011; Lee *et al.* 2012). In particular, the structure of the *Cannabis* endosperm was like that of the plants. This type of endosperm acts as a mechanical barrier to inhibit embryonic growth, and as a nutrient reserve for seed germination and early seedling establishment (Yan *et al.* 2014). In the later stage of germination, endosperm rupture and radicle protrusion occur through the seed coat, completing the germination process (Weitbrecht *et al.* 2011; Rajjou *et al.* 2012). Therefore, the structure and strength of the seed coats and pericarp are critical to controlling seed germination in many plants.

### Protein body and oil body in Cannabis seeds

The storage compounds are morphologically and biochemically remodelled extensively during germination (Waschatko *et al.* 2016). Protein bodies are vacuoles filled with storage proteins (Gunning and Steer 1996; Herman and Larkins 1999), and all storage proteins are initially synthesized on the rER (Bollini and Chrispeels 1979; Chrispeels 1991). Huang (1992, 1996) observed that lipase was newly synthesized *de novo* on free polysomes and bound specifically to the oil bodies during the germination of maize kernel. Additionally, the recognition signal on the oil bodies for lipase seemed to be captured by the oleosins.

In this study, the most prominent components found in *Cannabis* seeds were the protein bodies and oil bodies. A protein body contains a highly electron-dense globoid and a heterogeneous matrix and is surrounded by numerous oil bodies. Within the *Cannabis* seed, the large protein bodies are spherical or oval and ranged from 2.5 to 3.5 µm in diameter. Some researchers elucidated that 181 proteins were identified in hemp seeds with the main storage proteins globulin edestin in a concentration of 67–75 % and globular albumin that ranged from 25 to 37 % (Aiello *et al.* 2016; Aluko 2017). These proteins are antioxidants and act in a defensive role in germinating seeds when cleaved into fragments (Cattaneo *et al.* 2021). The protein content was within the range of 0.6 to 3.4 % (*w*/*w*) reported for oil bodies from seeds of various species (Tzen *et al.* 1993).

The most widespread sites for lipid accumulation in plant organs are seeds because high energy input is necessary for germination and seedling establishment (Penfield *et al.* 2004). Many plants store lipids in subcellular organelles, such as lipid droplets or oil bodies (Chapman *et al.* 2012; Song *et al.* 2017). LDs are surrounded by a monolayer and the surface-bound proteins are localized to the phospholipid monolayer (Walther *et al.* 2017). These organelles can protect the lipid reserves against oxidation and hydrolysis until seed germination and seedling establishment. Oil bodies are often considered to be spherical to ovoid, with diameters varying between species, ranging from 0.5 to 2.5 µm (Tzen *et al.* 1992; Wang *et al.* 2012). However, a study showed that the close packing of oil bodies in the cell matrix made them appear asymmetrical (Garcia *et al.* 2021).

In all plant seeds, oil bodies are found primarily in their cotyledons and radicles (Yoshida *et al.* 2003). They provide a source of energy for β-oxidation in neighbouring glyoxysomes during initial seed germination (Graham 2008; D’Andrea 2016). In *Medicago truncatula* oil bodies were aligned around the protein bodies (Song *et al.* 2017). However, many oil bodies were randomly filled with protein bodies in the cotyledon cells of *Cannabis*.

### Emulsifying proteins and stabilization of oil bodies

Various proteins are integrated into the lipid droplet monolayer or attached directly to the LD surface (Gidda *et al.* 2016; Huang 2018). The oil bodies are surrounded by a phospholipid monolayer and associated regulatory proteins and emulsifying proteins called oleosin (Huang 1994; Graham 2008; Horn *et al.* 2013; Pyc *et al.* 2017a; b). The presence of oleosin at the interface provides oil bodies with steric hindrance, protecting oil bodies from coalescence or aggregation (Huang 1992, 1994). For example, destroying the surface portions of the oleosins by tryptic digestion induces the coalescence of oleosomes and reveals severe changes in their adsorption kinetics (Tzen and Huang 1992).

Oleosin is associated with small oil bodies, whereas very large oil bodies lack oleosins and are stabilized by the lipid-associated proteins LDAP1 and LDAP2 (Horn *et al.* 2021). Interestingly, depending on the presence of oleosin, the oil bodies are variable in size. Oil bodies are very large from 10 to 20 μm in diameter when lacking oleosin, whereas oil bodies in the seed containing oleosin are 0.5 to 2 μm in diameter as seen in the mesocarp of avocado and olive (Ross *et al.* 1993).

The storage lipids in *Cannabis* seeds were stabilized by specific structural proteins, such as oleosin and caleosin that act as natural emulsifiers (Purkrtova *et al.* 2008). Garcia *et al.* (2021) reported the isolated oil bodies of hemp seed showed a uniform distribution of phospholipids and proteins at their interface. In this research, oil bodies in cotyledon cells of *Cannabis* appeared spherical and were measured ranging from 0.8 to 2 µm in diameter. These were extremely stable either inside the cells or in isolated preparations. Oil bodies inside the cells of mature seeds did not cluster or coalesce before germination.

Some membrane proteins function as effective emulsifying agents due to the presence of non-polar regions on their surfaces, which facilitate adsorption to oil–water or air–water interfaces (Sim *et al.* 2021). Indeed, it has been proven that oil bodies coalesced after following the proteolysis of surface oleosins (Maurer *et al.* 2013). However, how the oil bodies keep their small size without coalescing is not well known.

In a study by Gao and Goodman (2015), the interaction between LDs and other organelles, including ER, protein bodies, peroxisomes and mitochondria, was proven to occur through attachment between membranes. Although oleosin has a major influence on oil body size and distribution and maintains the integrity of the oil body in desiccation, seipen is another protein that is important in determining the number and size of oil bodies. Seipen in plants was discovered as homologs of animal and yeast seipen (Cai *et al.* 2015).

### rER-associated proteins as a trigger for the degeneration of oil bodies

Oleosins are degraded prior to lipid mobilization from oil bodies via ubiquitination–proteasome pathway (Deruyffelaere *et al.* 2015). Molecular studies frequently reveal intimate connections between LDs with the ER (Brocard *et al.* 2017; Choi *et al.* 2022). Both oil bodies and protein bodies of cotyledonary cells rapidly undergo morphological changes during germination, as they are utilized as a primary energy source. This process involved the degeneration of the organelles and their fusion with one another, resulting in irregularly contoured surfaces.

Although peroxisomes or glyoxysomes were not observed in the cotyledon cells of this research, rER was frequently observed in all cotyledon cells when germination began. Moreover, highly electron-dense substances appeared adjacent to oil bodies and attached to them. Similarly, the peroxisome-associated lipase translocates to the oil body surface to break down the stored lipids during seedling establishment (Thazar-Poulot *et al.* 2015). Such different occurrences of specific organelles in germinating cells of seeds imply that the degradation pathway of storage compounds has diverse types depending on the species and its developmental process.

We have found that the substances surrounding the oil bodies play a critical role in facilitating their degeneration during seed germination. These substances condense the contents of the oil bodies, recruit them to the half-membranes of oil bodies, and significantly enhance their conjugating activity. We hypothesize that specific proteins are synthesized in the rER and exported into the cytoplasm near the oil bodies. These proteins then translocate into the oil bodies through the half-membrane of oil bodies. They may act as enzymes with active sites on the surface region of oil bodies, leading to the weakening of the half-membranes and inducing them to coalesce. Therefore, rER-associated proteins act as a trigger in the degeneration mechanism of oil bodies, the oil bodies lose their morphology and fuse with each other. Finally, irregular hyaline areas are distributed throughout oil bodies, reflecting the destabilization of the emulsification of the oil bodies. Further studies on the degeneration of oil bodies in other species will provide data on the seed germination mechanisms of oily seed plants.

## Conclusions

As the storage organelles, protein bodies and oil bodies were packed in the cotyledon cells of *Cannabis* seeds. They remarkably changed the morphology at the early stage of germination. The storage proteins were concentrated in the centre of the protein body as a dense homogenous circular mass surrounded by a light heterogeneous area. Some of the storage proteins appear to act as emulsifying agents on the surface region of oil bodies. These proteins maintain the individuality and stability of oil bodies inside or outside the cotyledon cells. After rER appeared near the oil bodies, dense substances considered proteins rapidly aggregated adjacent to the oil bodies, resulting in the coalescence of oil bodies. We concluded that these rER-associated proteins play a key role in the degeneration of oil bodies by weakening the emulsifying agent on their non-polar surfaces and inducing the coalescence of oil bodies during seed germination. Finally, most oil bodies fused with one another and had an irregularly contoured surface at the late stage of germination.

## Data Availability

The data underlying this article are available in the article.
