# Correction to: Degeneration of oil bodies by rough endoplasmic reticulum (rER)-associated protein during seed germination in *Cannabis sativa* L.

**DOI:** 10.1093/aobpla/plae006

**Published:** 2024-02-12

This is a correction to: Eun-Soo Kim, Joon-Hee Han, Kenneth J Olejar, Sang-Hyuck Park, Degeneration of oil bodies by rough endoplasmic reticulum (rER)-associated protein during seed germination in *Cannabis sativa* L., AoB PLANTS, Volume 15, Issue 6, December 2023, plad082, https://doi.org/10.1093/aobpla/plad082

The originally published version of this manuscript has been corrected.

The title has been corrected to read “Degeneration of oil bodies by rough endoplasmic reticulum (rER)-associated protein during seed germination in *Cannabis sativa* L.”

In addition, figure legends have been updated with information regarding abbreviations and arrows.

